# Structure–Activity Relationship of Halophenols as a New Class of Protein Tyrosine Kinase Inhibitors

**DOI:** 10.3390/ijms12096104

**Published:** 2011-09-19

**Authors:** Xiu E. Feng, Wan Yi Zhao, Shu Rong Ban, Cheng Xiao Zhao, Qing Shan Li, Wen Han Lin

**Affiliations:** 1 School of Pharmaceutical Science, Shanxi Medical University, Taiyuan 030001, Shanxi, China; E-Mails: xiuefeng@163.com (X.E.F.); zhaowanyi@yahoo.cn (W.Y.Z.); shurongban@163.com (S.R.B.); zhaochengxiao1982@163.com (C.X.Z.); 2 School of Public Health, Shanxi Medical University, Taiyuan 030001, Shanxi, China; 3 State Key Laboratory of Natural and Biomimetic Drugs, Peking University, Beijing 100083, China; E-Mail: whlin@bjmu.edu.cn

**Keywords:** protein tyrosine kinase, halophenol, structure-activity relationship, benzophenone, diphenylmethane

## Abstract

A series of new benzophenone and diphenylmethane halophenol derivatives were prepared. Their structures were established based on ^1^H NMR, ^13^C NMR and HRMS data. All prepared compounds were screened for their *in vitro* protein tyrosine kinase (PTK) inhibitory activities. The effects of modification of the linker, functional groups and substituted positions at the phenyl ring on PTK inhibitory activity were investigated. Twelve halophenols showed significant PTK inhibitory activity. Among them, compounds **6c**, **6d**, **7d**, **9d**, **10d**, **11d** and **13d** exhibited stronger activities than that of genistein, the positive reference compound. The results gave a relatively full and definite description of the structure–activity relationship and provided a foundation for further design and structure optimization of the halophenols.

## 1. Introduction

Protein tyrosine kinases (PTKs), which are members of a large family of oncoproteins and proto-oncoproteins, play a major role in mitogenic signal transduction, and are involved in the control of cell proliferation, differentiation and transformation. Continuing activation of PTK is associated with proliferative disorders such as cancer; hence, PTK inhibitors have been developed as molecular-targeting cancer therapeutic agents. The discovery and development of PTK inhibitors as new cancer therapeutic agents have now attracted much attention [1–9]. To date, many PTK inhibitors with potent activities have already passed or are currently in clinical trials to investigate their applicability as anti-cancer drugs [10].

Various structural halophenols isolated from biologically active natural products such as various marine algae, ascidians and sponges present a wide spectrum of bioactivities including protein tyrosine phosphatase (PTP1B) inhibitory [11], antioxidative [12,13], antithrombotic [14], antimicrobial [15,16], anti-inflammatory [17], enzyme inhibitory [18], cytotoxic [19], and appetite suppressant [20], PTK inhibitory activities [21]. However, to the best of our knowledge, very little is known about the inhibitory activity of benzophenone and diphenylmethane halophenols against PTK, and their corresponding structure–activity relationships (SARs) have been rarely reported, despite the fact that several natural diphenylmethane bromophenols isolated from the brown alga *Leathesia nana* have been reported to show moderate inhibitory activity against PTK with over-expression of *c-kit* [21], which reveals that diphenylmethane halophenols may possess potential significant PTK inhibitory activity.

We therefore designed and synthesized a series of new diphenylmethane and benzophenone halophenol derivatives by modification of the linker (illustrated in Table 1), functional groups, and substituted positions at the phenyl ring to find novel structural halophenol derivatives with strong PTK inhibitory activity, and tried to establish the SAR on the basis of this new compound library. In our previous study [22], a series of bromo- and chloro- substituted halophenols were reported for their significant *in vitro* antioxidative and cytoprotective activities. However, the PTK inhibitory activity has not been evaluated. In the continued efforts towards discovering potent PTK inhibitors, a series of new fluoro- and iodo- functionalized benzophenone and diphenylmethane halophenols derivatives were also prepared and screened for their *in vitro* PTK inhibitory activity with genistein as positive control [23,24], in accordance with the fact that inclusion of F or I atoms in a compound may have profound effects on drug disposition [25–30]. The results provide some clear and useful information about recognition of the SAR.

## 2. Results and Discussion

### 2.1. Chemistry

**Scheme 1:**
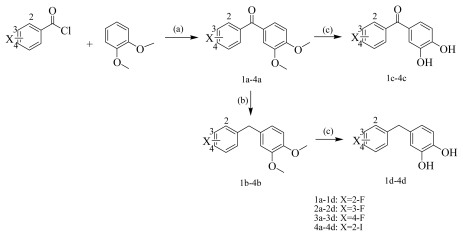
Synthesis of benzophenone and diphenylmethane halophenol derivatives. Reagents and conditions: (**a**) AlCl_3_/CH_2_Cl_2_, rt, 4 h; (**b**) LiAlH_4_/AlCl_3_/ether, 40 °C, refluxed for 2 h; (**c**) BBr_3_/CH_2_Cl_2_(1/9, v/v) was added to the solution at −78 °C, warmed to room temperature and stirred for 2 h.

A series of new benzophenone and diphenylmethane halophenol derivatives with several groups substituted at different positions on the phenyl ring were synthesized to determine how the substituents affect the PTK inhibitory activity. Halophenol compounds **5c-13c** and **5d-13d** were prepared in our previous study [22]. In the present study, 12 fluoro- and four iodo- functionalized halophenol derivatives (**1a-4d**) were also prepared (Scheme 1), including 11 new compounds **1b**, **1c**, **1d**, **2b**, **2c**, **2d**, **3b**, **3d**, **4b**, **4c** and **4d**.

### 2.2. *In vitro* PTK Inhibitory Activity

The *in vitro* PTK inhibitory activity of the prepared compounds listed in Table 1 was tested by ELISA with genistein as a positive reference compound. As shown in Table 1, 12 halophenols exhibited strong activities, which in some cases, were identical to, or even higher than, that of genistein in the same model. Among these, seven compounds, **6c**, **6d**, **7d**, **9d**, **10d**, **11d** and **13d**, showed the strongest activities with IC_50_ values of 2.97–12.9 μM, which were stronger than that of genistein with an IC_50_ value of 13.6 μM. Compound **8d** with an IC_50_ value of 14.8 μM exhibited identical activity to genistein. Compounds **8c**, **9c** and **11c** showed lower activities with IC_50_ values of 17.7, 17.8 and 16.0 μM, respectively. Compound **10c** exhibited weak activity with an IC_50_ of 41.6 μM.

### 2.3. SAR Analysis

Diphenylmethane halophenols **7d**, **8d**, **9d**, **10d**, **11d** and **13d** displayed higher activities with IC_50_ values of 6.34, 14.8, 12.9, 6.97, 6.26 and 5.05 μM than those of corresponding benzophenone halophenols **7c**, **8c**, **9c**, **10c**, **11c** and **13c**. Bromophenols **7c** and **13c**, which are isomers, showed no activity. Isomers of chlorophenols **8c**, **9c**, **10c** and bromophenol **11c** exhibited moderate activity with IC_50_ values of 17.7, 17.8, 41.6 and 16.0 μM, respectively. Replacement of the methylene group by a carbonyl group, except **6c** which showed similar activity to **6d**, led to an obvious decrease, even complete disappearance of the activity, which suggested that the methylene group may greatly contribute to the PTK inhibitory activity.

Meanwhile, substitution of the hydroxyl groups by methoxyl groups resulted in the disappearance of activity, and indeed, none of the compounds with methoxyl groups on the phenyl ring showed any activity with IC_50_ value higher than 50 μM. This indicated that the methoxyl group exerted a great negative effect on the PTK inhibitory activity, and also illustrated that the hydroxyl groups were essential. It is implied that these active halophenols as hydrogen donors could have key interactions with PTK.

By comparing the activities of the halogen-substituted compounds **5c**, **6c**, **5d** and **6d,** which possessed five hydroxyls and two halogen atoms at the same positions, we found that the chlorophenol compounds **6c** and **6d** exhibited the strongest activities with IC_50_ values of 2.97 μM and 3.96 μM, respectively. However, the bromophenols **5c** and **5d** showed no activity. Moreover, for all of the fluoro- and iodo- functionalized halophenols, no activity was observed. Hence, the halogen atoms on the phenyl ring contributed to the activity in the order of Cl > Br > F (or I), which suggested that the chloro atom may play a pivotal role between the interaction of active halophenols and PTK. The results also showed that an increased number of hydroxyl groups and chloro atoms may be beneficial to the activity.

Compounds **8c** and **9c** with a chloro atom at the ortho- and meta- position of the carbonyl group exhibited moderate activities, with IC_50_ values of 17.7 μM and 17.8 μM, respectively. Compound **10c** with a chloro atom at the para-position of the carbonyl group showed weak activity, with an IC_50_ value of 41.6 μM. Compounds **10d** and **9d**, with a chloro atom at the para- and meta- position of the methene group, showed high activities with IC_50_ values of 6.97 μM and 12.9 μM, respectively. Compound **8d** with a chloro atom at the ortho-position of the methene group exhibited identical activity with an IC_50_ value of 14.8 μM, compared to that of the positive control compound (IC_50_ = 13.6 μM). To the isomers of these chlorophenols, the chloro atom substituted at different position on the phenyl ring had significant effects on the activity. In addition, the same substituted position of the chloro atom on the phenyl ring had an entirely different effect on the activity of benzophenone and diphenylmethane halophenols. The results indicated that the linker and halogen had a combined influence on the activity.

The activities of compounds **12c** and **12d** were not enhanced by the increased number of halogen atoms. The bromophenols **11c** and **11d** substituted by one bromo atom obviously showed better activities than those of bromophenols **12c** and **12d** with two bromo atoms at the same phenyl ring.

## 3. Experimental Section

### 3.1. General

Melting points were taken on a micromelting point apparatus, which were uncorrected. ^1^H and ^13^C NMR spectra were recorded with a Bruker-AV 400 spectrometer at 400 and 100 MHz respectively, in CDCl_3_, DMSO-*d*_6_ or CD_3_OD with TMS as reference. Chemical shifts (δ values) and coupling constants (*J* values) were given in ppm and Hz, respectively. ESI mass spectra were obtained on an API QTRAP 3200 MS spectrometer, and HRMS were recorded on a Bruker Daltonics Apex IV 70e FTICR-MS (Varian 7.0T).

Ether was distilled from sodium benzophenone ketyl. Dichloromethane was distilled from calcium hydride. Other reagents and solvents were commercially available unless otherwise indicated.

### 3.2. Typical Procedures for the Preparation of Halophenol Derivatives

The general procedures for compounds **5c–13c**, and **5d–13d** have been reported in our earlier study [22]. A series of new fluoro- and iodo- functionalized compounds were prepared according to the following general procedures.

#### 3.2.1. Preparation of compounds **1a–4a**

##### 2-fluoro-3′,4′-dimethoxyl benzophenone (**1a**)

2-Fluorobenzoyl chloride (10 mL, 82.2 mmol) was added at 0 °C to a solution that contained 1,2-dimethoxybenzene (10 mL, 78.5 mmol) in dried CH_2_Cl_2_ (60 mL). Anhydrous AlCl_3_ (11.1 g, 83.2 mmol) was added by portion wise. The mixture was allowed to warm to room temperature and stirred for 4 h, then ice-water (100 mL) was added to the mixture. The organic phase was separated, washed with water (50 mL) and dried over anhydrous Na_2_SO_4_. The solvent was evaporated under reduced pressure, and the crude product was recrystallized from methanol to give compound **1a** as a white powder in 73% yield. m.p. 77–79 °C; ^1^H NMR (CDCl_3_) δ: 7.58 (d, 1H, Ph-6-H), 7.15–7.55 (m, 5H, PhH), 6.90 (d, 1H, Ph-5′-H), 3.97 (s, 3H, OCH_3_), 3.96 (s, 3H, OCH_3_); ^13^C NMR (CDCl_3_) δ: 192.0, 161.0, 158.5, 153.8, 149.1, 132.5, 132.4, 130.5, 130.4, 130.3, 127.5, 127.4, 125.9, 124.2, 124.1, 116.3, 116.1, 111.0, 109.9, 56.1, 56.0; MS (ESI) *m/z*: 261.0 ([M + H]^+^, 100).

Compounds **2a–4a** were prepared in the similar manner as described for **1a**.

##### 3-fluoro-3′,4′-dimethoxyl benzophenone (**2a**)

White powder, Yield 80%, mp 103–105 °C; ^1^H NMR (CDCl_3_) δ: 7.46–7.56 (m, 4H, Ph-2,4,5,6-H), 7.39 (dd, *J* = 2.0, 8.4 Hz, 1H, Ph-6′-H), 7.26–7.30 (m, 1H, Ph-2′-H), 6.93 (d, *J* = 8.4 Hz, 1H, Ph-5′-H), 3.96 (s, 3H, OCH_3_), 3.98 (s, 3H, OCH_3_); ^13^C NMR (CDCl_3_) δ: 194.1, 163.6, 161.2, 153.3, 149.2, 140.4, 140.3, 129.9, 129.8, 129.6, 125.6, 125.4, 118.9, 118.7, 116.6, 116.4, 112.0, 109.8, 56.1, 56.1; MS (ESI) *m/z*: 261.0 ([M + H]^+^, 100).

##### 4-fluoro-3′,4′-dimethoxyl benzophenone (**3a**)

White powder, Yield 86%, mp 101–103 °C; ^1^H NMR (CDCl_3_) δ: 7.80–7.84 (m, 2H, Ph-2,6-H), 7.48 (d, *J* = 2.0 Hz, 1H, Ph-2′-H), 7.37 (dd, *J* = 2.0, 8.4 Hz, 1H, Ph-6′-H), 7.15–7.19 (m, 2H, Ph-3,5-H), 6.93 (d, *J* = 8.4 Hz, 1H, Ph-5′-H), 3.98 (s, 3H, OCH_3_), 3.96 (s, 3H, OCH_3_); ^13^C NMR (CDCl_3_) δ: 194.2, 166.3, 163.8, 153.1, 149.1, 134.4, 132.3, 132.2, 130.1, 125.2, 115.4, 115.2, 112.1, 109.8, 56.1, 56.0; MS (ESI) *m/z*: 261.0 ([M + H]^+^, 100).

##### 2-iodo-3′,4′-dimethoxyl benzophenone (**4a**)

White powder, Yield 85%, mp 143–145 °C; ^1^H NMR (CDCl_3_) δ: 7.93 (d, *J* = 8.0 Hz, 1H, Ph-3-H), 7.60 (d, *J* = 2.0 Hz, 1H, Ph-6-H), 7.45 (m, 1H, Ph-5-H), 7.31 (dd, *J* = 2.0, 8.0 Hz, 1H, Ph-4-H), 7.16–7.21 (m, 2H, Ph-2′,6′-H), 6.86 (d, *J* = 8.4 Hz, 1H, Ph-5′-H), 3.96 (s, 3H, OCH_3_), 3.95 (s, 3H, OCH_3_); ^13^C NMR (CDCl_3_) δ: 196.1, 154.0, 149.4, 144.7, 139.5, 130.9, 128.6, 128.2, 127.7, 126.8, 111.1, 109.9, 92.4, 56.2, 56.1; MS (ESI) *m/z*: 369.0 ([M + H]^+^, 100).

#### 3.2.2. The Preparation of Compounds **1b–4b**

##### 2-fluoro-3′,4′-dimethoxyl diphenylmethane (**1b**)

Anhydrous AlCl_3_ (2.00 g, 15.0 mmol) and LiAlH_4_ (1.00 g, 26.4 mmol) were slowly added to dried ether (50 mL). After stirring for 5 min, compound **1a** (2.00 g, 7.69 mmol) was dissolved in dried ether (10 mL) and dropwisely added to the solution. The mixture was warmed to 40 °C, refluxed for 2 h, then cooled and quenched with 50 mL 18% aqueous HCl. The organic phase was separated. The water layer was extracted twice with ethyl acetate (2 × 30 mL). The combined organics were washed to neutral with water, dried over anhydrous Na_2_SO_4_, and then concentrated via rotary evaporation. The crude product was purified by silica gel chromatography with ethyl acetate-petroleum ether (1/9) as eluent to afford compound **1b**. The product was recrystallized from methanol to give a white powder in 40% yield. mp 51–52 °C; ^1^H NMR (CDCl_3_) δ: 7.04–7.24 (m, 4H, Ph-3,4,5,6-H), 6.77–6.84 (m, 3H, Ph-2′,5′,6′-H), 3.97 (s, 2H, CH_2_), 3.88 (s, 3H, OCH_3_), 3.87 (s, 3H, OCH_3_); ^13^C NMR (CDCl_3_) δ: 162.1, 159.7, 149.0, 147.5, 132.4, 130.9, 130.8, 128.4, 128.3, 127.9, 127.8, 124.1, 124.0, 120.8, 115.4, 115.2, 112.1, 111.2, 55.9, 55.8, 34.4; MS (ESI) *m/z*: 247.0 ([M + H]^+^, 100).

Compounds **2b-4b** were prepared in the similar manner as described for **1b**.

##### 3-fluoro-3′,4′-dimethoxyl diphenylmethane (**2b**)

Yellow liquid, Yield 62%; ^1^H NMR (CDCl_3_) δ: 7.26–7.30 (m, 1H, Ph-5-H), 7.01 (d, 1H, *J* = 7.2 Hz, Ph-6-H), 6.89–6.94 (m, 2H, Ph-2, 4-H), 6.85 (d, 1H, *J* = 8.0 Hz, Ph-5′-H), 6.72–6.77 (m, 2H, Ph-2′,6′-H), 3.95 (s, 2H, CH_2_), 3.89 (s, 3H, OCH_3_), 3.87 (s, 3H, OCH_3_); ^13^C NMR (CDCl_3_) δ: 164.2, 161.8, 149.0, 147.6, 144.1, 144.0, 132.8, 129.9, 129.8, 124.4, 121.0, 115.8, 115.5, 113.1, 112.9, 112.2, 111.3, 55.9, 55.8, 41.2; MS (ESI) *m/z*: 247.0 ([M + H]^+^, 100).

##### 4-fluoro-3′,4′-dimethoxyl diphenylmethane (**3b**)

Yellow liquid, Yield 40%; ^1^H NMR (CDCl_3_) δ: 7.15–7.18 (m, 2H, Ph-2,6,-H), 6.98–7.02 (m, 2H, Ph-3,5-H), 6.84 (d, 1H, *J* = 8.0 Hz, Ph-5′-H), 6.71–6.75 (m, 2H, Ph-2′,6′-H), 3.93 (s, 2H, CH_2_), 3.89 (s, 3H, OCH_3_), 3.86 (s, 3H, OCH_3_); ^13^C NMR (CDCl_3_) δ: 162.6, 160.2, 149.0, 147.5, 137.1, 137.0, 133.5, 130.2, 130.1, 120.8, 115.3, 115.1, 112.1, 111.3, 55.9, 55.8, 40.6; MS (ESI) *m/z*: 247.0 ([M + H]^+^, 100).

##### 2-iodo-3′,4′-dimethoxyl diphenylmethane (**4b**)

White powder, Yield 23%, mp 48–49 °C; ^1^H NMR (CDCl_3_) δ: 7.90 (dd, *J* = 1.2, 8.0 Hz, 1H, Ph-3-H), 7.27–7.31 (m, 1H, Ph-5-H), 7.13 (dd, *J* = 1.2, 7.6 Hz, 1H, Ph-6-H), 6.91–6.96 (m, 1H, Ph-4-H), 6.82 (d, *J* = 8.0 Hz, 1H, Ph-5′-H), 6.72–6.74 (m, 2H, Ph-2′,6′-H), 4.06 (s, 2H, CH_2_), 3.89 (s, 3H, OCH_3_), 3.86 (s, 3H, OCH_3_); ^13^C NMR (CDCl_3_) δ: 149.0, 147.6, 143.9, 139.5, 132.1, 130.2, 128.4, 128.0, 121.1, 112.4, 112.2, 101.2, 55.9, 55.8, 46.2; MS (ESI) *m/z*: 355.0 ([M + H]^+^, 100).

#### 3.2.3. The Preparation of Compounds **1c–4c**

##### 2-fluoro-3′,4′-dihydroxyl benzophenone (1c)

Ten milliliters BBr_3_ solution (BBr_3_/CH_2_Cl_2_, v/v, 1/9) was dropwisely added to a cooled (−78 °C) solution of compound **1a** (500 mg, 1.92 mmol) in dried CH_2_Cl_2_ (20 mL). The mixture was warmed to room temperature, stirred for 2 h, and poured into ice-water (50 mL). The organic phase was separated. The water layer was extracted twice with ethyl acetate (2 × 20 mL). The combined organics were washed with 20 mL water, dried over anhydrous Na_2_SO_4_ and concentrated via rotary evaporation. The crude product was purified by silica gel chromatography with ethyl acetate-petroleum ether (1/4, containing 1 mL acetic acid in 100 mL elution solvent) as eluent to afford compound **1c** as a white powder in 53% yield. mp 149–150 °C; ^1^H NMR (DMSO-*d_6_*) δ: 7.58–7.63 (m, 1H, Ph-6-H), 7.46–7.50 (m, 1H, Ph-4-H), 7.32–7.38 (m, 2H, Ph-3,5-H), 7.26 (d, 1H, *J* = 1.6 Hz, Ph-2′-H), 7.12 (dd, 1H, *J* = 1.2, 8.4 Hz, Ph-6′-H), 6.87 (d, 1H, *J* = 8.4 Hz, Ph-5′-H), 3.40 (br, 2H, Ph-OH); ^13^C NMR (DMSO-*d_6_*) δ: 191.5, 160.4, 158.0, 152.1, 145.8, *Int. J. Mol. Sci.* **2011**, *12* **6111** 132.9, 132.8, 130.3, 130.2, 128.8, 128.1, 127.9, 125.1, 125.0, 123.9, 116.6, 116.4, 115.8; MS (ESI) *m/z*: 231.0 ([M − H]^−^, 100); HRMS (ESI): Calcd. for C_13_H_9_F_1_O_3_ [M − H]^−^: 231.0463; Found: 231.0463.

Compounds **2c-4c** were prepared in the similar manner as described for **1c**.

##### 3-fluoro-3′,4′-dihydroxyl benzophenone (**2c**)

White powder, Yield 48%, mp 104–106 °C; ^1^H NMR (DMSO-*d_6_*) δ: 7.44–7.59 (m, 4H, Ph-2,4,5,6-H), 7.27 (d, 1H, *J* =2.0 Hz, Ph-2′-H), 7.15 (dd, 1H, *J* = 2.0, 8.0 Hz, Ph-6′-H), 6.89 (d, 1H, *J* = 8.4 Hz, Ph-5′-H), 5.00 (br, 2H, Ph-OH); ^13^C NMR (DMSO-*d_6_*) δ: 193.4, 163.4, 161.0, 151.5, 145.7, 141.8, 141.2, 141.1, 131.0, 130.9, 128.1, 125.6, 124.1, 117.3, 115.7; MS (ESI) *m/z*: 231.0 ([M − H]^−^, 100); HRMS (ESI): Calcd. for C_13_H_9_F_1_O_3_ [M − H]^−^: 231.0463; Found: 231.0463.

##### 4-fluoro-3′,4′-dihydroxyl benzophenone (**3c**)

White powder, Yield 55%, mp 155–157 °C; ^1^H NMR DMSO-*d*_6_) δ: 7.73–7.76 (m, 2H, Ph-2,6-H), 7.35–7.39 (m, 2H, Ph-3,5-H), 7.24 (d, 1H, *J* = 2.0 Hz, Ph-2′-H), 7.11 (dd, 1H, *J* = 2.0, 8.0 Hz, Ph-6′-H), 6.87 (d, 1H, *J* = 8.0 Hz, Ph-5′-H), 3.34 (br, 2H, Ph-OH); ^13^C NMR (CD_3_OD) δ: 194.3, 164.0, 158.1, 146.0, 133.4, 133.3, 133.2, 125.2, 123.1, 118.0, 116.4, 116.2, 115.8; MS (ESI) *m/z*: 231.0 ([M − H]^−^, 100); HRMS (ESI): Calcd. for C_13_H_9_F_1_O_3_ [M − H]^−^: 231.0463; Found: 231.0464.

##### 2-iodo-3′,4′-dihydroxyl benzophenone (**4c**)

White powder, Yield 60%, mp 186–188 °C; ^1^H NMR (DMSO-*d_6_*) δ: 7.89–7.95 (m, 1H, Ph-3-H), 7.46–7.54 (m, 1H, Ph-6-H), 7.16–7.31 (m, 2H, Ph-4,5-H), 7.00 (dd, 1H, *J* = 1.6, 8.4 Hz, Ph-6′-H), 6.77 (s, 1H, Ph-2′-H), 6.41 (d, 1H, *J* = 8.4 Hz, Ph-5′-H); ^13^C NMR (CD_3_OD) δ: 198.4, 153.2, 146.7, 146.6, 140.6, 131.9, 130.4, 129.0, 128.9, 126.2, 117.7, 116.0, 92.8; MS (ESI) *m/z*: 339.0 ([M − H]^−^, 100); HRMS (ESI): Calcd. for C_13_H_9_I_1_O_3_ [M − H]^−^: 338.9524; Found: 338.9514.

#### 3.2.4. The Preparation of Compounds **1d–4d**

##### 2-fluoro-3′,4′-dihydroxyl diphenylmethane (**1d**)

To a cooled (−78 °C) solution of compound **1b** (400 mg, 1.63 mmol) in dried CH_2_Cl_2_ (5 mL) was dropwisely added 5 mL BBr_3_ solution (BBr_3_/CH_2_Cl_2_, v/v, 1/9). The mixture was warmed to room temperature, stirred for 15 min, and poured into ice-water (50 mL). The organic layer was separated. The water layer was extracted twice with ethyl acetate (2 × 10 mL). The combined organics were washed with 10 mL water and dried over anhydrous Na_2_SO_4_, then concentrated via rotary evaporation. The crude product was purified by silica gel chromatography with ethyl acetate/petroleum ether (3/7, containing 1 mL acetic acid in 100 mL elution solvent) as eluent to afford compound **1d** as pale yellow powder in 40% yield. mp 91–92 °C; ^1^H NMR (DMSO-*d_6_*) δ: 8.80 (dd, 1H, *J* = 4.8, 41.6 Hz, Ph-6-H), 7.10–7.27 (m, 3H, Ph-3,4,5-H), 6.67 (d, 1H, *J* = 8.0 Hz, Ph-6′-H), 6.59 (s, 1H, Ph-2′-H), 6.50 (d, 1H, *J* = 8.0 Hz, Ph-5′-H), 3.79 (s, 2H, CH_2_), 3.42 (br, 2H, Ph-OH); ^13^C NMR (DMSO-*d*_6_) δ: 162.0, 159.5, 145.6, 144.1, 131.7, 131.6, 131.1, 129.1, 128.9, 128.5, 128.4, 124.8, 124.7, 119.7, 116.4, 116.0, 115.7, 115.5, 33.9; MS (ESI) *m/z*: 217.0 ([M − H]^−^, 100); HRMS (ESI): Calcd. for C_13_H_11_F_1_O_2_ [M − H]^−^: 217.0670; Found: 217.0672.

Compounds **2d–4d** were prepared in the similar manner as described for **1d**.

##### 3-fluoro-3′,4′-dihydroxyl diphenylmethane (**2d**)

Pale yellow powder, Yield 66%, mp 66–68 °C; ^1^H NMR (DMSO-*d_6_*) δ: 7.28–7.34 (m, 1H, Ph-5-H), 6.99–7.04 (m, 3H, Ph-2,4,6-H), 6.68 (d, 1H, *J* = 8.0 Hz, Ph-5′-H), 6.60 (d, 1H, *J* = 2.0 Hz, Ph-2′-H), 6.51 (dd, 1H, *J* = 2.0, 8.0 HZ, Ph-6′-H), 3.78 (s, 2H, CH_2_), 3.42 (br, 2H, Ph-OH); ^13^C NMR (DMSO-*d_6_*) δ: 163.9, 161.5, 145.6, 145.5, 145.4, 144.1, 131.9, 130.6, 130.5, 125.1, 119.9, 116.6, 116.0, 115.7, 115.5, 113.1, 112.9, 40.5; MS (ESI) *m/z*: 217.0 ([M − H]^−^, 100); HRMS (ESI): Calcd. for C_13_H_11_F_1_O_2_ [M − H]^−^: 217.0670; Found: 217.0672.

###### 4-fluoro-3′,4′-dihydroxyl diphenylmethane (**3d**)

White powder, Yield 59%, mp 59–61 °C; ^1^H NMR (DMSO-*d_6_*) δ: 7.32–7.86 (m, 2H, Ph-2,6-H), 6.95–7.23 (m, 2H, Ph-3,5-H), 6.66 (d, 1H, *J* = 8.0 Hz, Ph-5′-H), 6.55 (d, 1H, *J* = 2.0 Hz, Ph-2′-H), 6.45 (dd, 1H, *J* = 2.0, 8.0 Hz, Ph-6′-H), 3.88 (s, 2H, CH_2_), 3.38 (br, 2H, Ph-OH); ^13^C NMR (DMSO-*d*_6_) δ: 162.3, 159.9, 145.6, 143.9, 138.6, 138.5, 132.5, 130.8, 130.7, 119.8, 116.5, 116.0, 115.5, 115.3, 40.0; MS (ESI) *m/z*: 217.0 ([M − H]^−^, 100); HRMS (ESI): Calcd. for C_13_H_11_F_1_O_2_ [M − H]^−^: 217.0670; Found: 217.0672.

###### 2-iodo-3′,4′-dihydroxyl diphenylmethane (**4d**)

White powder, Yield 40%, mp 130–132 °C; ^1^H NMR (DMSO-*d_6_*) δ: 7.86 (dd, 1H, *J* = 1.2, 8.0 Hz, Ph-3-H), 7.36 (m, 1H, Ph-5-H), 7.23 (dd, 1H, *J* = 1.6, 7.6 Hz, Ph-6-H), 6.99 (m, 1H, Ph-4-H), 6.66 (d, 1H, *J* = 8.0 Hz, Ph-5′-H), 6.55 (d, 1H, *J* = 2.0 Hz, Ph-2′-H), 6.47 (dd, 1H, *J* = 2.0, 8.0 Hz, Ph-6′-H), 3.88 (s, 2H, CH_2_), 3.38 (br, 2H, Ph-OH); ^13^C NMR (DMSO-*d_6_*) δ: 144.4, 139.6, 130.9, 130.8, 128.9, 128.7, 120.0, 116.5, 116.4, 116.0, 115.9, 101.8, 45.3; MS (ESI) *m/z*: 325.0 ([M − H]^−^, 100); HRMS (ESI): Calcd. for C_13_H_11_I_1_O_2_ [M − H]^−^: 324.9731; Found: 324.9726.

### 3.3. Pharmacology

After removal of the male mouse (SD) meninges, the brain was weighted and homogenized with a glass homogenizer in four volumes of cold medium (containing 20 mmol/L Tris–HCl, pH 7.5, 0.25 mol/L sucrose, 2 mmol/L DDT, 2 mmol/L EDTA, 2 mmol/L Na_3_VO_4_, 1 mmol/L DMSF, 215 mg/L aprotinin, 1 mg/L PTIFtatin, 5 mg/L leupeptin) at high speed. The mixture was centrifuged for 10 min at 1000 × g at 4 °C; the supernatant was collected and re-centrifuged for 10 min at 10,000 × g at 4 °C to obtain the target supernatant. The target supernatant that contained cytoplasmic tyrosine kinase was collected, separately packed and stored at −70 °C.

PTK activity was determined by the ELISA method. In brief, the concentrations of PTKs used to construct calibration curves were as follows: 600, 500, 341, 200 and 100 × 10^−7^ U/mL for PTK. 100 μL of 20 mg/L PGT (Sigma Aldrich) in 20 mmol/L PBS solution was added to 96-well microtiter plates at 37 °C overnight. After removing excess substrate solution, PBS–Tween 20 (PBST) was used to wash the wells one time, which were dried for 2 h at 37 °C and kept at 4 °C. Subsequently, 50 μL of the above PTK extraction (50 mmol/L HEPES, pH 7.4, 20 mmol/L MgCl_2_, 0.1 mmol/L MnCl_2_, 0.2 mmol/L Na_3_VO_4_, 0.6 mmol/L ATP) was added to the 96-well plates. Then, a certain concentration of the tested compounds (10 μL) and tyrosine kinase tissue extract solution were incubated at 37 °C for 1 h. The plate was washed three times with PBST at the end of the treatment, and 100 μL of the diluted horseradish-peroxidase-labeled mouse anti-phosphotyrosine monoclonal antibody IgG_2bk_ (Sigma Aldrich) was added to each well, and incubated at 37 °C for 30 min. After removal of the antibody complex, and then washing three times with PBST, 100 μL freshly prepared TMB horseradish peroxidase color development solution (Beijing 4A Biotech Co., Ltd, China) was added and protected from light for 10 min. The reaction was terminated by addition of 100 μL/well 1 N sulfuric acid. Absorbance was measured at 450 nm in a microplate reader (SpectraMax M5) [4].

## 4. Conclusions

In this study, we prepared a series of new benzophenone and diphenylmethane halophenol derivatives and investigated the influence of the linker, substituted groups, number and positions of halogen atoms on PTK inhibitory activities.

All of the chlorophenols exhibited strong activities, and the substituted position of the chloro atom also markedly affected the activity. Unfortunately, all of the fluoro- and iodo- functionalized compounds were inactive. Our findings confirmed that the hydroxyls and halogens (chloro or bromo) seem to be essential to the activity, and the chloro atom and methylene group play a crucial role in the activity. Hence, these compounds with high activities represent a new paradigm for halophenols and appear to offer great potential as new PTK inhibitors. The results also gave a relatively full recognition of the SARs, and provided a foundation for further structural optimization of the halophenols in the search for suitable clinical candidates.

## Figures and Tables

**Table 1:** Structures and *in vitro* protein tyrosine kinase (PTK) inhibitory activities of the prepared halophenols. PTK activity was determined by the ELISA method with genistein as positive control.

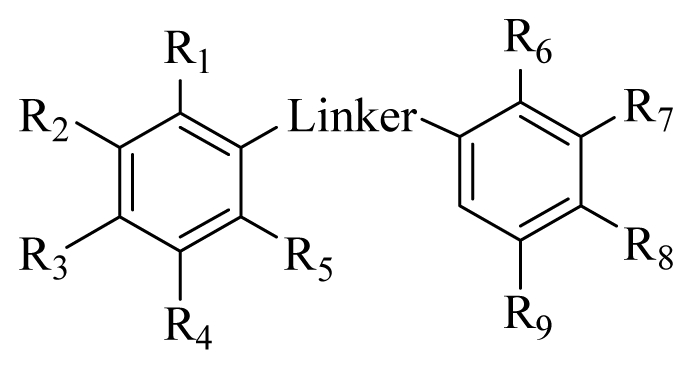											
Compd.	Substituted group										IC_50_^a^ (μM)
	Linker	R_1_	R_2_	R_3_	R_4_	R_5_	R_6_	R_7_	R_8_	R_9_	
1c *	C=O	F	H	H	H	H	H	H	OH	OH	>50
1d *	CH_2_	F	H	H	H	H	H	H	OH	OH	>50
2c *	C=O	H	F	H	H	H	H	H	OH	OH	>50
2d *	CH_2_	H	F	H	H	H	H	H	OH	OH	>50
3c	C=O	H	H	F	H	H	H	H	OH	OH	>50
3d *	CH_2_	H	H	F	H	H	H	H	OH	OH	>50
4c *	C=O	I	H	H	H	H	H	H	OH	OH	>50
4d *	CH_2_	I	H	H	H	H	H	H	OH	OH	>50
5c	C=O	Br	OH	OH	OH	Br	H	H	OH	OH	>50
5d	CH_2_	Br	OH	OH	OH	Br	H	H	OH	OH	>50
6c	C=O	Cl	OH	OH	OH	Cl	H	H	OH	OH	2.97
6d	CH_2_	Cl	OH	OH	OH	Cl	H	H	OH	OH	3.96
7c	C=O	H	Br	OH	H	H	Br	H	OH	OH	>50
7d	CH_2_	H	Br	OH	H	H	Br	H	OH	OH	6.34
8c	C=O	Cl	H	H	H	H	H	H	OH	OH	17.7
8d	CH_2_	Cl	H	H	H	H	H	H	OH	OH	14.8
9c	C=O	H	Cl	H	H	H	H	H	OH	OH	17.8
9d	CH_2_	H	Cl	H	H	H	H	H	OH	OH	12.9
10c	C=O	H	H	Cl	H	H	H	H	OH	OH	41.6
10d	CH_2_	H	H	Cl	H	H	H	H	OH	OH	6.97
11c	C=O	H	H	H	H	H	Br	H	OH	OH	16.0
11d	CH_2_	H	H	H	H	H	Br	H	OH	OH	6.26
12c	C=O	H	H	H	H	H	Br	Br	OH	OH	>50
12d	CH_2_	H	H	H	H	H	Br	Br	OH	OH	>50
13c	C=O	OH	H	H	Br	H	Br	H	OH	OH	>50
13d	CH_2_	OH	H	H	Br	H	Br	H	OH	OH	5.05
Control (Genistein)											13.6

* New compound;

a The IC_50_ values were determined in triplicate.
